# *In Vitro* Ultrastructural Changes of MCF-7 for Metastasise Bone Cancer and Induction of Apoptosis via Mitochondrial Cytochrome C Released by CaCO_3_/Dox Nanocrystals

**DOI:** 10.1155/2014/391869

**Published:** 2014-06-16

**Authors:** Abdullahi Shafiu Kamba, Maznah Ismail, Tengku Azmi Tengku Ibrahim, Zuki Abu Bakar Zakaria, Lawal Hassan Gusau

**Affiliations:** ^1^Laboratory of Molecular Biomedicine, Institute of Bioscience, Universiti Putra Malaysia (UPM), 43400 Serdang, Malaysia; ^2^Faculty of Veterinary Medicine, Universiti Putra Malaysia (UPM), 43400 Serdang, Malaysia; ^3^Department of Pure and Applied Chemistry, Usmanu Danfodiyo University, PMB, Sokoto 2346, Nigeria

## Abstract

Bones are the most frequent site for breast cancer cells to settle and spread (metastasise); bone metastasis is considered to have a substantial impact on the quality of patients with common cancers. However, majority of breast cancers develop insensitivity to conventional chemotherapy which provides only palliation and can induce systemic side effects. In this study we evaluated the effect of free Dox and CaCO_3_/Dox nanocrystal on MCF-7 breast cancer using MTT (3-[4, 5-dimethylthiazol-2-yl]-2, 5-diphenyl tetrazolium bromide), neural red, and lactate dehydrogenase colorimetric assays while DNA fragmentation and BrdU genotoxicity were also examined. Apoptogenic protein Bax, cytochrome C, and caspase-3 protein were analysed. Morphological changes of MCF-7 were determined using contrast light microscope and scanning and transmission electron microscope (SEM and TEM). The findings of the analysis revealed higher toxicity of CaCO_3_/Dox nanocrystal and effective cells killing compared to free Dox, morphological changes such as formation of apoptotic bodies, membrane blebbing, and absent of microvilli as indicated by the SEM analysis while TEM revealed the presence of chromatin condensation, chromosomal DNA fragmentation, cell shrinkage, and nuclear fragmentation. Results of TUNEL assay verified that most of the cells undergoes apoptosis by internucleosomal fragmentation of genomic DNA whereas the extent of apoptotic cells was calculated using the apoptotic index (AI). Therefore, the biobased calcium carbonate nanocrystals such as Dox carriers may serve as an alternative to conventional delivery system.

## 1. Introduction

Bone is one of the most common sites of metastasis in individuals with breast cancer. The most common complication of breast cancer is the ability to metastasise to distant locations with a special specification to bone. Therefore, more than 70% of breast cancer deaths are due to the colonisation of cancer cells in bone leading to metastasis at the secondary site [1]. Treatment for bone metastasis involves the use of combination of local and systemic therapy, which involves hormones, surgical management, and radiation therapy, resulting in the removal of cancer cells from the affected site and treatment of impending fractures [2]. The use of conventional chemotherapy, especially Doxorubicin, can inhibit the progression of many types of tumour, providing symptomatic relief and the regression of bone cancer [3]. However, the major drawback of chemotherapy is the lack of selectivity to cancer cells and an absence of specificity, thereby causing the development of multidrug resistance during prolonged treatment and less accumulation of the drug at its intracellular target site of action. This is due to the overexpression of efflux transporters such as P-glycoprotein (P-gp), which is the major mechanism of drug resistance in cancer [4, 5]. The design of nanoparticulate delivery systems that can accommodate drugs and other bone-targeting molecules can potentially overcome the drawbacks presented by conventional approaches (chemotherapy and radiation therapy) [3]. The major challenge in the field of nanomedicine is to develop an alternative nanomaterial that can meet the basic requirements for a therapeutic delivery system. Recently, the use of inorganic materials such as delivery carriers has emerged in the research field of nanotherapeutics, imaging, and diagnostic systems.

Calcium carbonate nanocrystals are among those novel inorganic materials with high potential in biomedical applications and can also act as carriers for a control delivery system due to their biodegradability, biocompatibility, and porous nature [6]. CaCO_3_ has turntable physicochemical properties, surface chemistry (shape and size), and easy method of production in a large scale [7]. Another advantage of CaCO_3_ nanocrystals is that they are largely pH sensitive: as the pH decreases, the solubility increases exponentially [8]. Thus, nanocarriers have received considerable attention in disease diagnosis and treatment with a specific example in cancer diagnostics and therapy; this is due to the unique characteristics and behaviour at the tumour site [9]. Nanoparticle-targeted anticancer delivery strategies act by evading the reticuloendothelial system (RES), thus effectively accumulating in the tumour via the enhanced permeability and retention (EPR) effect [9, 10]. Other advantages of the nanoparticle delivery system include the ability to protect the drug component from premature degradation [6].

To the best of our knowledge, no research has been reported concerning the use of bio-based calcium carbonate nanocrystals derived from cockle shells (*Anadara granosa*), in which the primary component is calcium carbonate (95–98%) [11]. Here, we are specifically interested in the synthesis of a rod-shaped aragonite polymorph of calcium carbonate for drug delivery applications. It was described by other researchers that rod-shaped nanocarriers have unique characteristic and are more selective and effective in cancer delivery systems than when compared with the spherical shape of the same material [12].

The main purpose of this study is to examine the mechanism of cellular pathway which can either be necrotic or apoptotic cell death through ultrastructural changes induced by the effect of doxorubicin delivered by CaCO_3_ nanocrystals.

## 2. Materials and Methods

### 2.1. Synthesis and Drug Loaded Calcium Carbonate Nanocrystals

The synthesis of calcium carbonate nanocrystals was carried out in oil-in-water (O/W) microemulsions using a higher pressure homogeniser (HPH) as described in our published articles [13, 14].

Drug loaded calcium carbonate nanocrystals were prepared according to Kamba et al. [14] with slight modification in the quantity drug used. Aqueous solution of Doxorubicin hydrochloride (2 mg/mL) was added into the calcium carbonate nanocrystals suspension (containing 50 mg of nanocrystals). The encapsulation of Dox in CaCO_3_ nanocrystals was achieved by continuous stirring of the suspension mixture in the dark overnight at room temperature. Calcium carbonate nanocrystals containing Dox were washed, centrifuged, and oven* dried* (FD 115, Fisher Scientific, Limburg, Germany) at 55°C.

### 2.2. Characterisation of Drug Calcium Carbonate Nanocrystals

The morphology and particle size of calcium carbonate nanocrystal were analysed by transmission electron microscopy (TEM, Hitachi H-7100,Tokyo, Japan) and field emission scanning electron microscopy (FESEM, JOEL 7600F, Tokyo, Japan) as described in our published article [14].

FTIR analysis, pellets of pure doxorubicin, CaCO_3_/Dox nanocrystals, and calcium carbonate nanocrystals were calibrated in a weight proportion of 1 wt% in Kbr, and analyses were performed with a Fourier-transform infrared spectrometer (FTIR, model 100, Perkin Elmer, 710 Bridgeport Avenue, Shelton, USA) in the range of 400 to 4000 cm^−1^.

### 2.3. Determination of Drug Loading Content and Encapsulation Efficiency

The drug loading and encapsulation efficiency in the CaCO_3_ nanocrystals were analysed by calculating the difference between the total of the drug feed (Wt) and the free drug (Wf) concentrations in the nanoparticle supernatant per weight of CaCO_3_ nanocrystals. The formulated mixture was centrifuged at 5000 g for 15 minutes and the amount of free Dox remaining in the supernatant of solution was determined by measuring the absorbance at 485 nm in a UV-vis spectrophotometer (PerkinElmer Lambda 35 Boston, MA, USA). Data were given as average measurements of 3 independent values. Drug loading and encapsulation efficiency are calculated according to [15] using

(1) $$\begin{array}{c} \text{Loading}\;\text{content}=\frac{\text{Wt}-\text{Wf}}{\text{WnP}}\times 100, \end{array}$$

where Wt is the total weight of drug fed, Wf is the weight of nonencapsulated free drug, and Wnp is the weight of the nanoparticle:

(2) $$\begin{array}{c} \text{Encapsulation}\;\text{efficiency}=\frac{\text{Wt}-\text{Wf}}{\text{Wt}}\times 100, \end{array}$$

where Wt is the total weight of drug fed and Wf is the weight of nonencapsulated free drug.

### 2.4. Cells Culture and* In Vitro* Evaluation of Cytotoxicity

Breast cancer cells MCF-7 were purchased from the American Type Culture Collection (ATCC, Manassas, VA, USA) and cultured in a DMEM medium supplemented with 10% foetal bovine serum, L-glutamine (15 mmol/L), penicillin (100 U/mL), and streptomycin (100 *μ*g/mL). The culture was incubated in a 5% CO_2_ atmosphere at 37°C. Cells at 80%–90% confluence were used for seeding and treatment.

### 2.5. *In Vitro* Evaluation of Cytotoxicity (Mitochondrial Activity)

The cells were seeded into a 96-well plate at a density of 1 × 10^5^ per well and incubated for 24 h. Cells were cocultured with different concentrations of doxorubicin and the CaCO_3_/Dox nanocrystals suspension (0 to 2 *μ*g/mL) for periods of 24, 48, and 72 h. After the exposure was completed, media were aspirated and washed with PBS before being replaced with additional media without serum prior to MTT treatment. Aliquots of 20 *μ*L MTT reagent (Sigma Aldrich) in PBS were added into each well and incubated at 37°C for 4 h (Thermo Fisher Scientific LPG). Then, the culture medium was removed and 200 *μ*L of dimethylsulphoxide (DMSO) was added into the wells. The plate was kept in a dark room for 30 minutes and optical densities of the solutions were measured at 570 nm, with a microplate reader being used to release the coloured product into solution. The experiments were conducted in triplicate.

### 2.6. Neutral Red (NR) Assay for Lysosomal Activity

Neutral red assay was conducted by the method and protocol of Repetto et al. [16]. The same procedure was used as in MTT. After exposure was completed, the media was aspirated and washed with PBS twice before replacement with supplemented 100 *μ*L neutral red reagents of 40 *μ*g/mL (Sigma Aldrich, St. Louis, US) in PBS to each well and the plate was incubated at 37°C for 2 h (Thermo Fisher Scientific LPG Hudson, New Hampshire, USA). After this incubation, the neutral red mediums were removed and washed with PBS, followed by addition of 150 *μ*L neutral red destain solution (mixture of 1% glacial acetic acid, 50% ethanol, and 49% distilled water) per well for 20 minutes to extract the neutral red dye. The plate optical densities of the solutions were measured at 540 nm. The experiments were conducted in triplicate.

### 2.7. BrdU Proliferation Assay

Cell proliferation was analysed based on the incorporation of BrdU (5-bromo-2′-deoxyuridine) into the synthesised DNA during cell proliferation. For BrdU analysis, similar procedure was conducted as indicated in MTT. After treatment, the cells were subjected to a 5-bromo-2-deoxyuridine labelling assay according to the manufacturer's protocols (Roche Diagnostics GmbH, Mannheim, Germany). The absorbance was measured at 550 nm using a microplate reader and the data was presented as mean ± SD.

### 2.8. DNA Fragmentation Assay

DNA fragmentation assays were analysed. The treated MCF-7 and control cells were centrifuged at 200 g for 10 min. The cells were lysed at room temperature for 20 min with 400 mL lysis solution comprised of 0.2% Triton X-100, 1 mM EDTA, and 10 mM Tris-HCl, at pH 7.5. To pellet the intact DNA, the lysates were centrifuged at 13,000 g. The supernatant containing the fragmented DNA was transferred to a separate tube. Additional lysis solution (400 mL) was added to the pellet of intact DNA. To precipitate the DNA, 200 mL of 25% trichloroacetic acid (TCA) was added, and the samples were incubated at 4°C overnight. The TCA was removed, and 80 mL of 5% TCA was added to the DNA to facilitate hydrolysis at 90°C for 10 min. Diphenylamine (DPA) reagent (160 mL), comprised of 1.5% sulphuric acid, 0.2% acetaldehyde, and 98% glacial acetic acid, was added to the supernatant and pellet samples, and they were then incubated at room temperature overnight for colour development by an unknown mechanism. The OD was determined using a microtitre plate reader. The percentage of DNA fragmented was calculated by the following equation:

(3) %  of  DNA  fragmentation =DNA  Supernantant  DNA  Pellet+Supernantant×100.

### 2.9. LDH Release (Membrane Integrity Assays)

MCF-7 breast cancer cells were seeded into 96-well plate at a seeding density of 1 × 10^5^ and incubated for 24 hrs. The medium was removed and replaced with CaCO_3_/Dox nanocrystal concentrations (0 to 2 *μ*g/mL). The plates were incubated 37°C for 24, 48, and 72 hrs. After incubation, 2 *μ*L of lysis buffer was added to the positive control wells, and the plate was centrifuged (Thermo Fisher Scientific LPG, Hudson, New Hampshire, USA) at 1,500 rpm for 10 min at 37°C. After centrifugation, 50 *μ*L of a membrane integrity assay reagent was added to the wells. The plates were incubated for 10 min at 37°C protected from light. Thirty (30 *μ*L) of HCl 1N stop reagent were added into the wells and the fluorescence of the samples was measured at 560 nm (excitation) and 590 nm (emission) on the microplate reader. The percentage of cytotoxicity was calculated with respect to the positive control wells whereby the lysed cells were assumed to have 100% lactate dehydrogenase (LDH) release.

### 2.10. Measurement of Caspase-3 Activity

Proteolytic activities of caspase-3 were measured by colorimetric assay kits (Sigma-Aldrich St. Louis, US) according to the manufacturer's instructions. Briefly, cells were seeded in a dish at a seeding density of 1 × 10^5^ and after reaching confluence; serum-containing medium was aspirated and cocultured with various concentrations of CaCO_3_/Dox in serum-free medium for 24 h. Cell pellets were suspended in a cell lysis buffer and incubated on ice for 10 min. The lysate was vortexes every 15 min. After centrifugation at 11,000 g for 15 min, 20 *μ*L of cell lysate were added immediately to buffer containing* p-*nitroaniline (pNA)-conjugated substrate for caspase-3 (Ac-DEVDpNA). The samples were incubated for 1 h at 37°C. Samples fluorescence was detected in a microplate reader (Spectra Fluor, TECAN, Sunrise, Austria). Concentration of the released pNA was calculated from the absorbance values at 405 nm and caspase activities are expressed as fold increases over the nontreated cells control.

### 2.11. Enzyme-Linked Immunosorbent Assay (ELISA Abnova HmbH, Heidelberg, Germany)

Bax and cytochrome C protein activity were evaluated. The cells were seeded at a density of 1 × 10^6^ per dish and incubated overnight at 37°C; then the cells were treated with various concentrations of CaCO_3_/Dox loaded nanocrystal and incubated for 24 h; total protein was extracted from the cells using lysis buffer-containing protease inhibitor cocktail set III and phosphatase inhibitor cocktail set I (Calbiochem, EMD Biosciences, San Diego, CA). The protein concentrations of all the samples were determined with A Bicinchoninic Acid Protein Assay Kit (Invitrogen Carlsbad, CA, USA). Cytochrome C was determined by Enzyme-Linked Immunosorbent Assay (ELISA Abnova HmbH, Heidelberg, Germany) and the levels of Bcl-2 and Bax proteins were quantified using the Human Bcl-2/Bax ELISA Kit, according to the manufacturer's instructions (Human Apoptosis regulator Bcl-2/bax; Cusabio, California, USA).

### 2.12. Microscopic Examination of Cellular Morphology

MCF-7 breast cancer cells were seeded in 6-well plates at a seeding density of (1 × 10^5^) incubated overnight for cells to adhere to dish surface. The cells were washed with PBS then the cells were exposed to CaCO_3_/Dox loaded nanocrystal for 12, 24, and 72 hr using IC_50_ concentrations (calculated from MTT results). Morphology and membrane changes were examined through light-inverted microscope.

### 2.13. Transmission Ultrastructural Effects of Doxorubicin Loaded Calcium Carbonate Nanocrystal on MCF-7 Breast Cancer

MCF-7 breast cancer cells were cocultured with both CaCO_3_ nanocrystal and CaCO_3_/Dox nanocrystal and incubated for 24, 48, and 72 hours at 37°C. The cultured cells were trypsinised and centrifuged for 10 minutes at 3500 rpm at room temperature. The pellets were fixed in 4% (v/v) glutaraldehyde in 0.1 M cacodylate buffer (pH 7.4) for 4 hours at 4°C. The fixed cells were centrifuged and the pellets were blocked in serum which was later fixed in 2.5% glutaraldehyde for 2 hours at 4°C. The specimens were washed in three changes of sodium cacodylate buffer (pH 7.4) for 10 minutes each, postfixed in 1% osmium tetroxide for 2 hours at 4°C. The samples were washed again in three changes of sodium cacodylate buffer (pH 7.4) for 10 minutes each and dehydrated in ascending grades of acetone (35%, 50%, 75%, 95%, and 100%). The cells were then infiltrated with acetone and resin and embedded with 100% resin in beam capsule, and left to polymerize at 60°C for 48 hours. The area of interest in the embedded cells resin block was chosen using the toluidine blue staining and later examined using light microscope. The selected area was cut in ultrathin sections using ultramicrotome. The sections were placed into a grid and stained with uranyl acetate for 10 minutes followed by 50% filtered acetone, and finally stained using lead which was then washed twice with distilled water. The stained samples were then viewed under transmission electron microscopy (Phillips, Eindhoven, and The Netherlands).

### 2.14. Scanning Electron Microscope (SEM)

MCF-7 breast cancer cells were cocultured with both CaCO_3_ nanocrystal and CaCO_3_/Dox and incubated for 24, 48, and 72 hours at 37°C. The cultured cells were trypsinised and centrifuged for 10 minutes at 3500 rpm at room temperature. The cells were washed twice with PBS (pH 7.4) and centrifuge for 10 min at 3000 rpm. The pellets were fixed in 4% (v/v) glutaraldehyde in 0.1 M cacodylate buffer (pH 7.4) for 4 hrs at 4°C. The fixed cells were washed in three changes of sodium cacodylate buffer for 10 minutes each, and postfixed in 1% osmium tetroxide at 4°C. The samples were then washed in three changes of sodium cacodylate buffer (pH 7.4) for 10 minutes each, dehydrated in ascending grades of acetone (35%, 50%, 75%, 95%, and 100%), and brought to critical point drying for thirty minutes. The cells were affixed to a metal SEM stub and sputter coated in gold by using an SEM coating unit (E5100 Polaron, UK). The coated specimens were viewed using SEM (JOEL 64000, Japan) at an accelerating voltage of 25 KV.

### 2.15. TUNEL Assay for Apoptotic DNA Fragmentation

To further authenticate and confirm the DNA fragmentation quantified using diphenylamine reagent (colorimetric assay) for the induction of apoptosis in MCF-7 by CaCO_3_/Dox nanocrystal. TUNEL assay was performed, after 24 h of cells culture; the cells were treated with CaCO_3_/Dox nanocrystal for 24, 48 and 72 h. DNA cleavage was verified using enzymatic end labelling of DNA strand breaks using a commercial kit (proTUNEL assay for apoptotic DNA Fragmentation Kit, GeneTex, Inc. (North America) 2456 Alton Pkwy Irvine, CA 92606 USA) according to the manufacturer's instructions before analysing by confocal laser scanning microscopy (Olympus confocal Microscope System Tokyo, Japan).

### 2.16. Statistical Analyses

All statistical analyses were performed using Minitab statistical software (Minitab Inc, State College, PA, USA) and Origin 8. Treatment effects were determined using one-way analysis of variance followed by Tukey's post-hoc analysis. A value of *P* < 0.05 was considered significant unless indicated otherwise.

## 3. Results and Discussion

**Figure 1:**
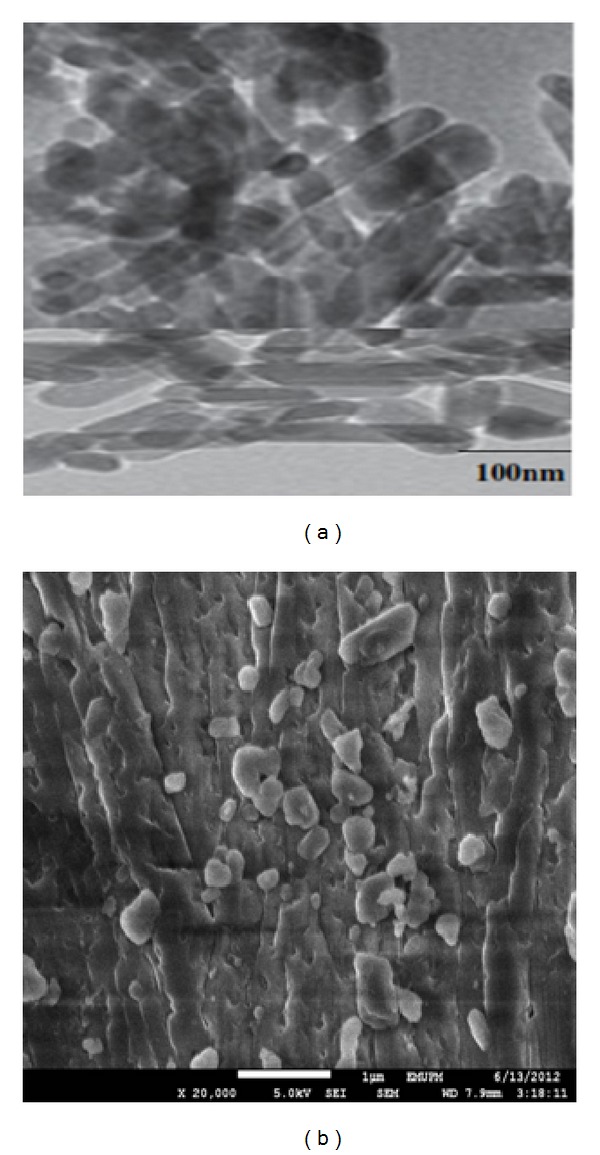
(a) TEM micrograph of the Dox-loaded rod-shape nanocrystals and (b) FESEM micrograph of CaCO_3_/Dox nanocrystals.

Figures 1(a) and 1(b) demonstrate micrograph images for TEM and FESEM for the synthesised drug calcium carbonate nanocrystals. The micrographs in Figures 1(a) and 1(b) reveal a rod-shape image of the synthesised CaCO_3_ loaded with doxorubicin. Detail characterisation of synthesised CaCO_3_/Dox nanocrystal was fully discussed in our previous article [14].

**Figure 2:**
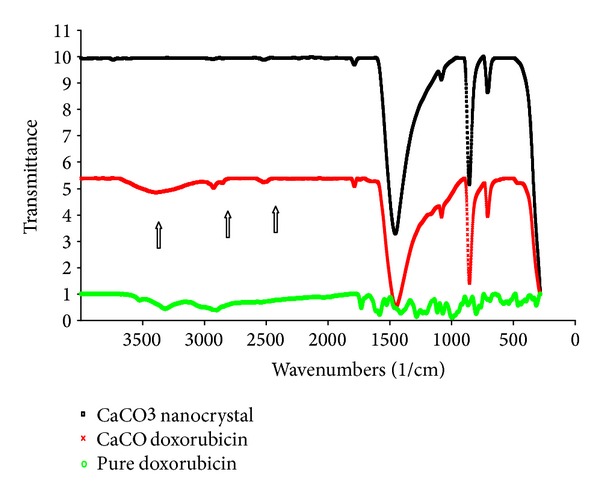
FTIR spectra of CaCO_3_ nanocrystal, CaCO_3_/Dox loaded nanocrystal, and free Dox.

Figure 2 display FTIR spectra of CaCO_3_ nanocrystal, CaCO_3_/Dox loaded nanocrystal and free Dox, the presence of Doxorubicin is confirmed by the peak (2925, 2896, 2537 and 3526) in CaCO_3_/Dox loaded nanocrystal resulting in broadening at the stated peaks compared with unloaded CaCO_3_ nanocrystal spectrum, similar peaks were also observed at the pure doxorubicin spectrum, therefore, it can be interpreted that the attachment or loading of Doxorubicin in CaCO_3_ nanocrystal was successfully achieved. However, the broad absorption peak of CO_3_^2−^ at ~1455 cm^−1^, ~1082 cm^−1^, ~1786 cm^−1^, ~855 cm^−1^ and ~709 cm^−1^, are reported to be the common characteristic features of the CO_3_^2−^ in CaCO_3_. Moreover, the observed bands at ~1082 cm^−1^ and ~855 cm^−1^ were carefully assigned as *v*_1_ symmetric stretching and *v*_2_ out-of-plane bending modes of CO_3_^2−^, respectively. The peak at ~1082 cm^−1^ was only observed in the spectrum of aragonite-phase calcium carbonate, whose CO_3_^2−^ ions are inactive in the infrared region.

### 3.1. Drug Loading and Encapsulation Efficiency

Drug loading capacity of CaCO_3_ nanocrystals may be due to the porous nature of the synthesised nanocrystals, as shown by FESEM in Figure 1(b). The loading content of Dox in CaCO_3_ was observed to be ~86% when 2 mg/mL of Dox was loaded to 50 mg of calcium carbonate nanocrystals. It was shown that the loading capacity has a tremendous effect on the encapsulation efficiency; this was observed in our previous studies when 1 mg/mL was loaded into 50 mg of calcium carbonate nanocrystals, the encapsulation efficiency was about 97%, whereas in this study, 2 mg/mL of Dox was loaded to 50 mg of the nanocrystal but the loading content was only 86%. This indicates that the higher the drug loading, the lower the drug encapsulation efficiency [14, 17].

### 3.2. Cytotoxicity Evaluation of Doxorubicin and CaCO_3_/Dox Nanocrystals (MTT Assay)

**Figure 3:**
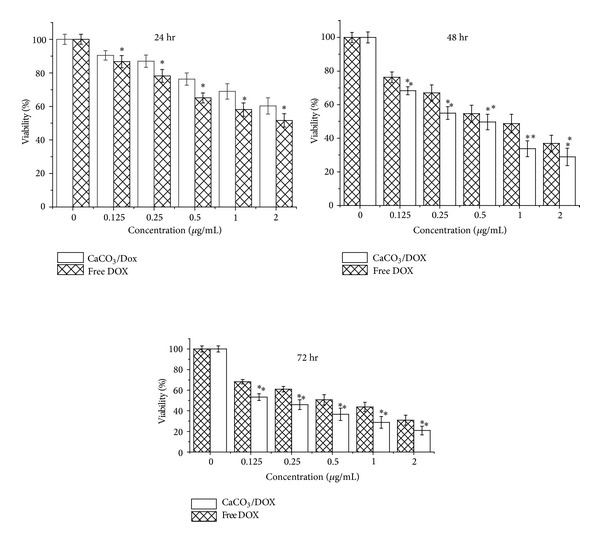
*In vitro* cytotoxicity study of MCF-7 cells for incubation periods of 24, 48, and 72 hr with free DOX and the CaCO_3_/Dox nanocrystals. ∗Means with different superscript are statistically significant *P* < 0.05 compared to CaCO_3_/Dox for 24 hr whereas ∗∗means with different superscript are statistically significant *P* < 0.05 compared to free Dox for 46 and 72 hr.

Figure 3 shows the cytotoxicity of free Dox and Dox-loaded calcium carbonate nanocrystals on the breast cancer cell line MCF-7. The cellular toxicity of the free Dox was significantly higher (*P* < 0.05) compared to Dox-loaded nanocrystals in the first 24 hr of treatments; the growth inhibition rates of both free Dox and Dox-loaded nanocrystals demonstrated a concentration and time-dependent toxicity. However, the higher toxicity of free doxorubicin to MCF-7 may be a result of direct contact of the drug with the cells which led to a considerable growth inhibition effect in MCF-7 cell line. The effect of CaCO_3_/Dox nanocrystals on the MCF-7 cell line was observed to have a lesser effect in the first 24 hr of treatment than the free Dox at the same concentration. The reason for this lesser effect may be due to the differences in cellular uptake mechanisms and drug release for both the free Dox and CaCO_3_/Dox, the effect of CaCO_3_/Dox was only effective at inhibiting MCF-7 after a considerable amount of Dox was released from the calcium carbonate nanocrystal. This was in contrast to the free Dox, which had direct contact with the MCF-7 cell line. The mechanism of CaCO_3_/Dox delivery to MCF-7 cells is non-specific endocytosis, and the delivery system mechanism may avoid the effect of multidrug resistance proteins, which are always present in cancer cells [18, 19].

The acidic pH increases the cellular uptake of weakly acidic drugs, thereby increasing the delivery effect of the drug to the target site. In contrast, retardation and weak uptake of basic drugs such as Doxorubicin are caused by acidic pH, resulting in reduced drug efficiency to the target of action [17].

### 3.3. Cytotoxicity Evaluation of Doxorubicin and CaCO_3_/Dox Nanocrystals (Neutral Red Uptake Assay)

**Figure 4:**
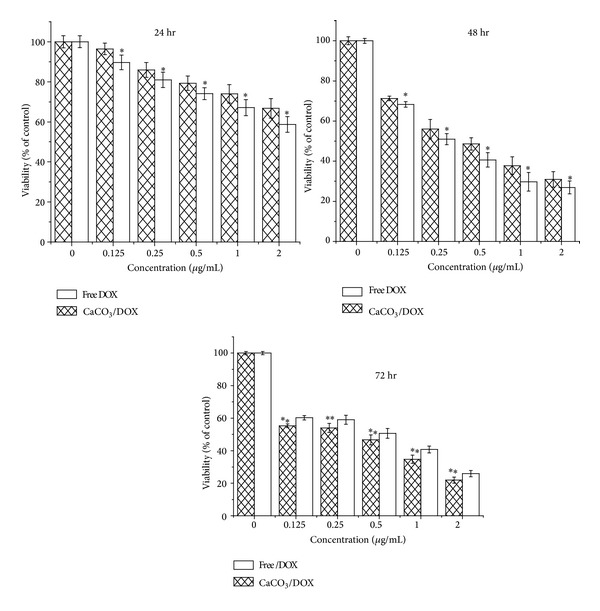
*In vitro* cytotoxicity study of MCF-7 cells for incubation periods of 24, 48, and 72 hr with free Dox and the CaCO_3_/Dox nanocrystals. ∗Means with different superscript are statistically significant *P* < 0.05 compared to CaCO_3_/Dox for 24 hr whereas ∗∗means with different superscript are statistically significant *P* < 0.05 compared to free Dox for 46 and 72 hr.

Figure 4 shows the cytotoxicity of free Dox and Dox-loaded calcium carbonate nanocrystals on breast cancer cell line MCF-7. The cellular toxicity of the free Dox was significantly higher (*P* < 0.05) compared to Dox-loaded nanocrystals for 24 and 48 hr with exception of 72 hr where rapid decrease in growth inhibition rates of MCF-7 cells was observed; this may be due to the fact that the effect of CaCO_3_/Dox was only effective at inhibiting the growth of MCF-7 after a considerable amount of Dox was released from the calcium carbonate nanocrystals at the 72 hr of treatment. However, both free Dox and Dox-loaded nanocrystals demonstrated a concentration- and time-dependent toxicity. The neutral red assay was not consistent with our present result in MTT; this may possibly be due to their sensitivity and mechanism of action; it was observed that MTT is more sensitive in the study of viability/toxicity compared to neutral red assay [20].

### 3.4. Cell Proliferation Assays (BrdU)

**Figure 5:**
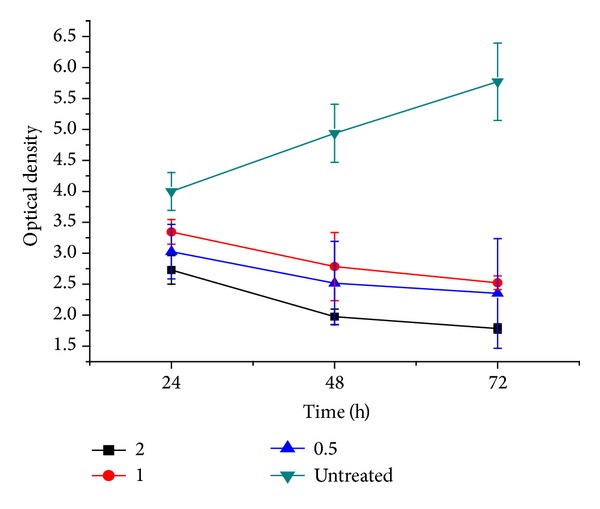
Antiproliferative description of CaCO_3_/Dox on MCF-7 cells (BrdU Assay).

To examine the antiproliferative effect of calcium carbonate nanocrystals containing Dox on MCF-7, the analysis was conducted by the addition of BrdU to the cell culture; the proliferating cells will incorporate this into their DNA just as they would incorporate thymidine. The cellular proliferation was evidently decreased after treatment with Dox-CaCO_3_ for the incubation periods of 24, 48, and 72 hr. The growth inhibition of MCF-7 was significantly reduced compared to the untreated group in a time-dependent manner, as indicated in Figure 5. The results of the BrdU assay were found to be similar to those reported for the NR assay. The ability of calcium carbonate nanocrystals loaded with Doxorubicin indicates a potential delivery system of the synthesised nanocrystals for anticancer drugs.

### 3.5. DNA Fragmentation Assay

**Figure 6:**
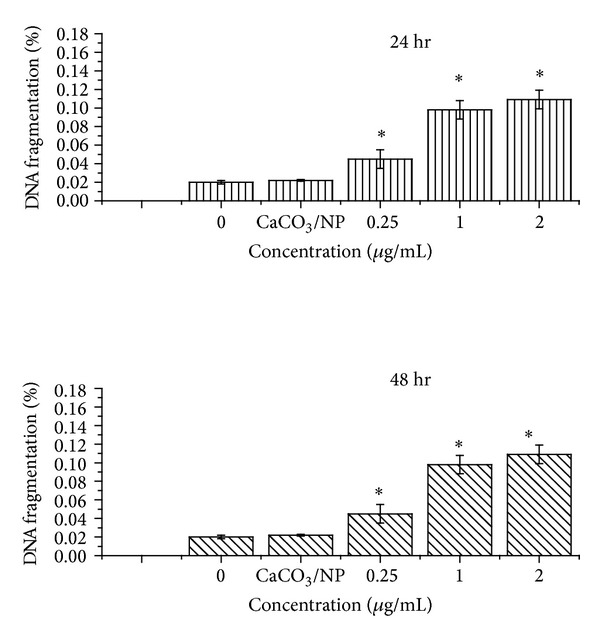
DNA fragmentation assay after treatment with CaCO_3_/Dox for 48 hr. The fragmented DNA was quantified using diphenylamine reagent (colorimetric assay). ∗Means with different superscript are statistically significant *P* < 0.05 compared to control and CaCO_3_/NP.

Fragmentation of cellular DNA is a distinctive characteristic of cellular nucleic acid undergoing apoptosis. The quantifications of DNA fragments after treatment with various concentrations of calcium carbonate nanocrystals loaded with the doxorubicin anticancer agent for the incubation period of 24 and 48 hr are presented in Figure 6. The effects of treatment on the relative quantity of fragmented DNA are observed to be dose and time-dependent. However, dose-dependent treatment showed significant differences in both 24 and 48 hr treatment groups, but only small differences were expressed in relation to the incubation time of 24 hr when compared with the control or untreated group.

The data presented in Figure 6 are an indication of accelerated Dox release at acidic pH, thereby intercalating the DNA and preventing the synthesis of nucleic acids. Such characteristics are believed to be the last event of apoptosis and are regarded as a hallmark of cell death [21].

### 3.6. LDH Release (Membrane Integrity Assay)

Measurement of LDH activity in the cell supernatant is one of the most popular methods for the assessment of* in vitro* cytotoxicity potential of a compound, extract, or formulation. Therefore, membrane integrity was analysed by measuring the amount of LDH released, which is proportional to the number of cells damaged or lysed.

**Figure 7:**
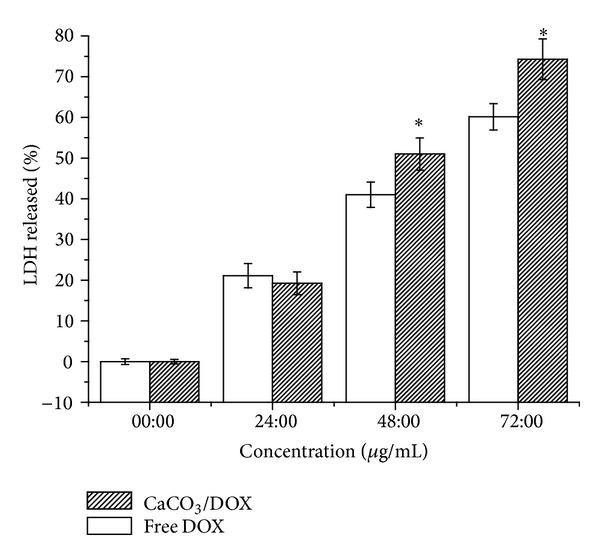
LDH released analysed for membrane integrity assay. ∗Means with different superscript are statistically significant *P* < 0.05 compared to control and free Dox for 48 and 72 hr.

The release of LDH from the supernatants of lysed MCF-7 cells after exposure was slightly higher in free DOX for the first 24 h (*P* < 0.05) compared to CaCO_3_/Dox nanocrystals within the same period, likely as a result of the slow release of Dox from the nanocrystal. However, the LDH release was observed to be higher for the periods of 48 and 72 hr after exposure to CaCO_3_/Dox nanocrystals, as indicated in Figure 7. Apparently, the total release of Dox was significantly higher in the loaded CaCO_3_ (CaCO_3_/Dox nanocrystals) than that of free Dox. This result agrees with BrdU assays (Figure 4), where by the same mechanism may be used to explain the increase in LDH released at 48 and 72 hr by the CaCO_3_/Dox nanocrystals.

### 3.7. Bax, Cytochrome C, and Caspase-3 Protein Expression in MCF-7

**Figure 8:**
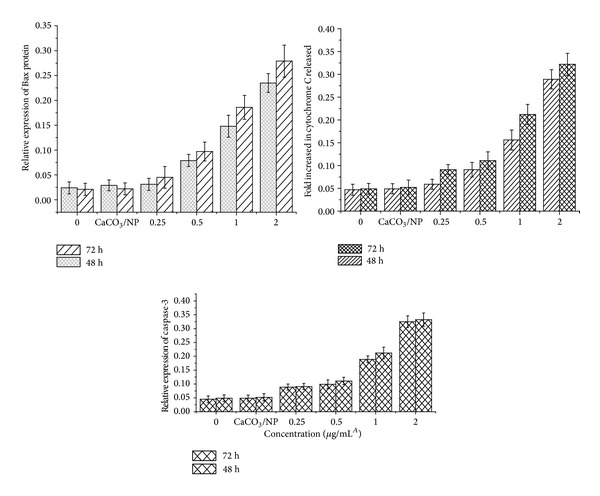
Translocation of cytochrome C into the cytosol, relative expression of Bax, and caspases 3 proteins.

Treatment of MCF-7 with CaCO_3_/Dox lead to the expression of apoptotic protein involved in cell death and signalling pathway, which was observed to be concentration and time dependent. Up regulation of proapoptotic member of Bcl2 family (Bax) protein and apoptotic executor caspase-3 was noticed as indicated in Figure 8. Selim and Hendi [22] observed that activation of Bax protein may provoke permeabilisation of outer mitochondria membrane and resulted in the release of a soluble protein from intermembrane space into the cytosol, thereby promoting caspase activation.

Released of cytochrome C by the cells was observed to be connected with the mitochondrial related apoptosis. However, the treatment of MCF-7 with CaCO_3_/Dox nanocrystal induced the release of cytochrome C, the release was found to be increasing in concentration and time dependent manner. Therefore, our result may suggest the possible death signalling pathway for MCF-7 via mitochondrial cytochrome C released.

### 3.8. Morphological Examinations by Contrast Light Microscope

**Figure 9:**
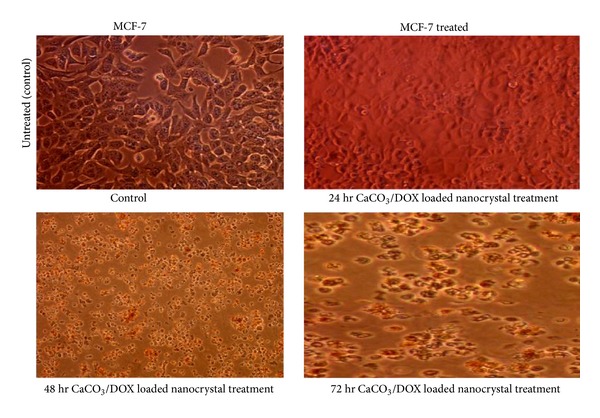
Inverted light microscope images of morphological changes on MCF-7 treated with Dox nanocrystals.

Morphological observation of MC-7 after treated with CaCO_3_/Dox nanocrystal as in Figure 9, the treated cells were looking unhealthy and dying out of populace in first 12 hr, the observed cells were more reduced and dying as treatment duration increases. Cells detachment, shrunken, and dispersed cells into the culture medium and floating cells were also observed. The observed morphological changes increases in sequential order with culture duration as showed in Figure 9. Whereas, control cells treated with only culture media despite the duration of treatment, the cells maintained most of their morphological features as seen in Figure 9.

### 3.9. Ultrastructural Changes in MCF-7 Breast Cancer Cells

Electron microscopy is one of the versatile analytical techniques used for the analysis of surface and morphological features in cancer cells therapy [23]. Advanced microscopic tools with higher resolutions such as the transmission electron microscope (TEM) and the scanning electron microscope are among the best equipment for the ultrastructural study and observation of cellular changes during apoptosis progression of cellular death, and it was also indicated to be the most appropriate method that can differentiate between the necrotic and apoptosis cell deaths [24, 25].

**Figure 10:**
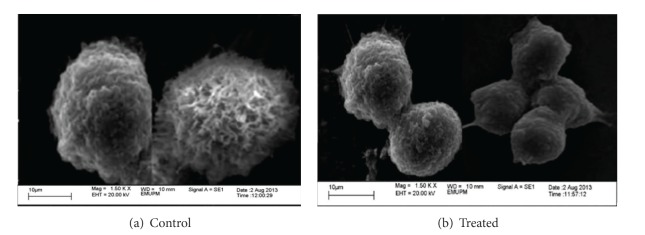
Control with no treatment (a) while (b) is treated with CaCO_3_ nanocrystal.

**Figure 11:**
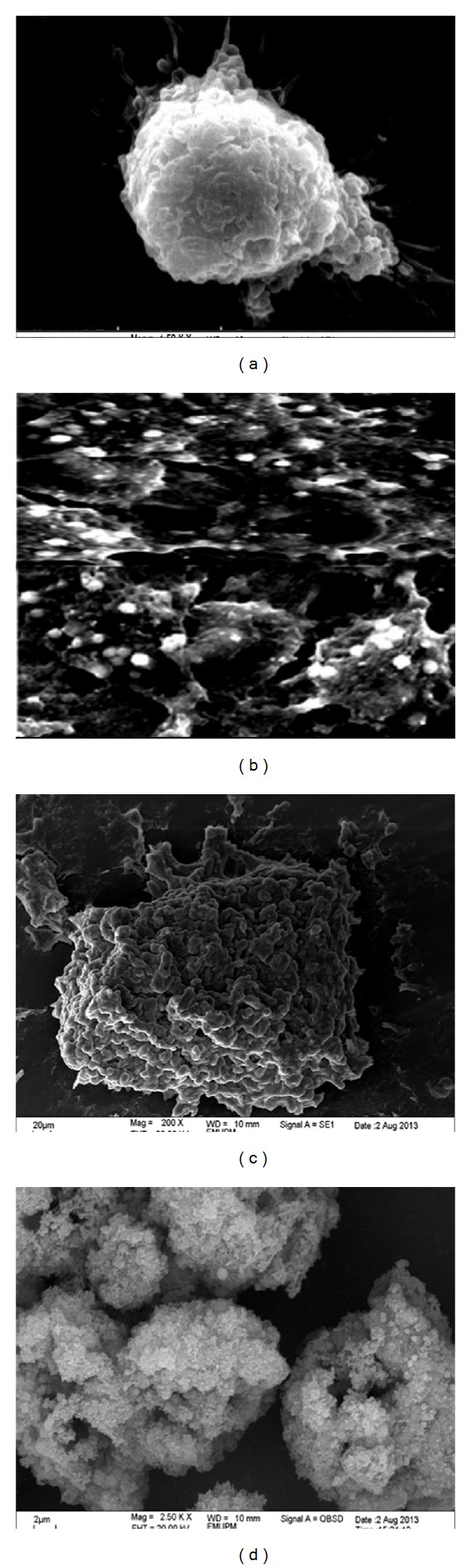
(a) is a control group without treatment while (b), (c), and (d) were treated with CaCO_3_/Dox nanocrystal for 24, 48, and 72 hr, respectively.

Characteristic morphological changes of breast cancer MCF-7 cells were observed by Scanning Electron Microscope as displayed in Figures 10 and 11. According to Figure 10(a) is a control group without treatment and observed to have a typical morphology of healthy cells that are well attached to the surface, possessing numerous microvilli and lamellipodia on the cell surfaces and intact cell membrane connection. In Figure 11(b), the cells were treated with CaCO_3_ nanocrystals, and the possible features observed were in similar trend identified in Figure 11(a) with a slight increase in the number of microvilli at the cell surfaces. It was reported that numerous microvilli on the cell surfaces are among the characteristic features of healthy cancer cells [26]. The presented morphological analysis has established that CaCO_3_ nanocrystals showed no toxicity to MCF-7 breast cancer cells. This was further indicated in our previous study [17].

The treated MCF-7 cells are demonstrated in Figure 11. The control group in Figure 11(a) shows well-defined cellular processes with good attachment on the surfaces, microvilli, and wider lamellipodia; whereas in the treated groups (see Figures 11(b), 11(c) and 11(d), significant morphological changes were exposed as seen in Figure 11. Cell clumping and resultant apoptotic bodies formation was apparent, and other features of morphological changes that can be witnessed in the treated groups 11(c) and 11(d), which showed typical morphological apoptotic bodies on the cell surfaces including membrane blebbing, as observed with a scanning electron microscope (SEM) presented in Figure 11(b).

**Figure 12:**
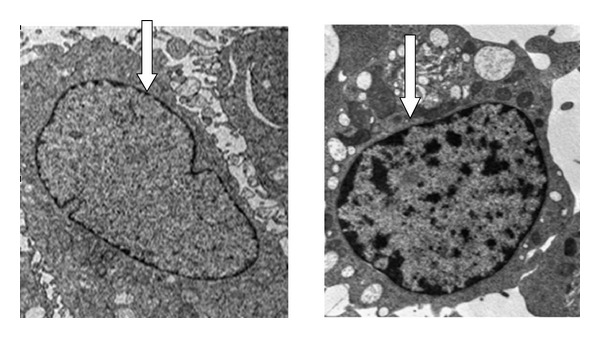
Transmission electromicrograph of untreated MCF-7 bone cancer displaying normal cellular process containing complete integrity of cells organelle and also showing rounded shape cell with nucleus and nucleolus chromatin. Image magnification ×10,000; bar 2 *μ*m.

**Figure 13:**
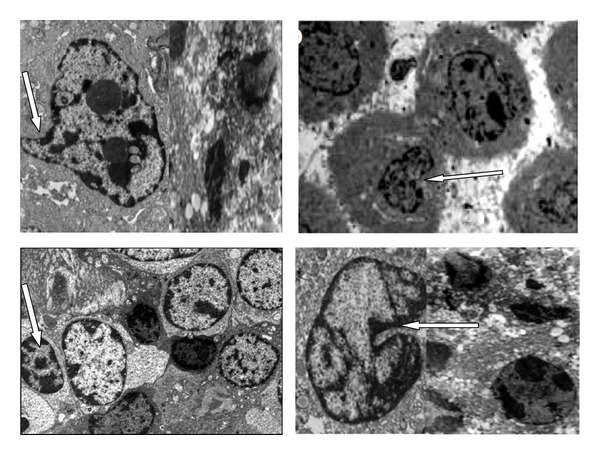
Ultrastructural changes of treated MCF-7 breast cancers showing features of early apoptosis: cell shrinkage and chromatin condensation and late apoptosis: nuclear collapse, progressing blebbing, and apoptotic body formation. Magnification ×10,000; bar 2 *μ*m.

Figures 12 and 13 demonstrate ultrastructural characteristic of untreated and treated MCF-7.  Figure 12 indicates a normal characteristic of cancer cells with complete cell organelle and well-distributed chromatin and a clear nuclear membrane.

The image in Figure 13 demonstrated some major sign of early apoptosis by reduction in cells size and appearing of cytoplasm condensation while maintaining complete membrane integrity and formation of smooth plasma membrane. The rounded cells become smaller and elongated which further break into smaller apoptotic bodies and appearances of nuclear blebs as shown in Figure 13. The apoptotic observable features in the treated MCF-7 breast cancer cells include cells shrinkage, chromomatin condensation, and apoptotic bodies with complete membrane has indicated that MCF-7 breast cancer cells undergoes cells death via apoptosis path way on contrary to untreated groups with well-preserved normal morphology in Figure 12.

### 3.10. Internucleosomal Degradation of DNA (TUNEL Assay)

**Figure 14:**
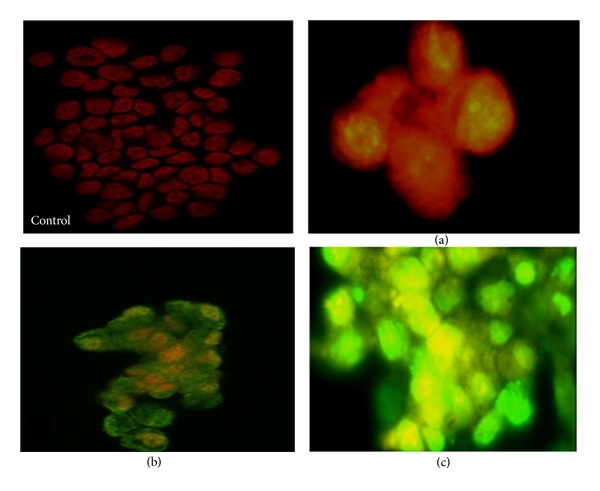
TUNEL DNA fragmentation assay on MCF-7 cells after treatment with Dox loaded CaCO_3_ nanocrystal. (a) is untreated cells showing complete absent of green fluorescence colour while (b), (c), and (d) are treated cells for incubation period of 24, 48, and 72 h, respectively. The cells showed depicting of TUNEL positive stain progressively according to the duration period.

To confirm and validate the DNA fragmentation quantified using diphenylamine reagent (colorimetric assay) for the induction of apoptosis in MCF-7 by CaCO_3_/Dox nanocrystal as indicated in Figure 6. Deoxyribonucleic acid (DNA) Fragmentation (TUNEL assay) was conducted and is presented in Figure 14. During programmed cell death, the chromosomal DNA is degraded by apoptotic endonucleases [26]. As indicated in Figure 14(a), all the cells were stained red showing the viability of the untreated MCF-7 cells as the control group. While the remaining groups (b), (c) and (d) were treated MCF-7 cells with Dox-loaded calcium carbonate nanocrystals, it was observed that the numbers of apoptotic cells were increasing in a time-dependent manner. Therefore a significant increase in the amount of fragmented DNA as time progressively increases from 24 to 72 hours is shown by the higher green fluorescence in the treated groups ((b), (c), and (d)).

**Figure 15:**
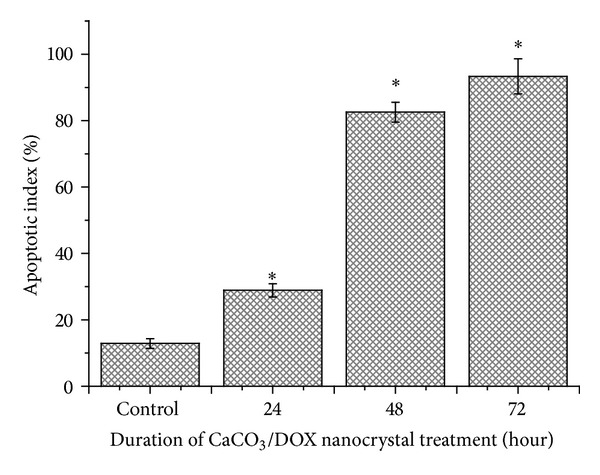
Apoptotic index MCF-7 cells treated with CaCO_3_/Dox nanocrystal. ∗Means with different superscript are statistically significant *P* < 0.05 compared to control.

Figure 15 shows the percentage apoptotic index of treated MCF-7 cells with CaCO_3_/Dox nanocrystals. It was demonstrated that the percentage of apoptotic cells increased in a time-dependent manner with less than 50% representation of apoptotic cells of the total cultured cells in 24 hours while at 48 hours approximately 80% of the cultured cells died via the apoptosis pathway.

## 4. Conclusion

In this study, we examined the effect of drug loading and cytotoxicity of doxorubicin delivery for targeting metastatic bone cancer using biobase calcium carbonate nanocrystal. We further provided morphological evidence to prove the cellular apoptotic pathway, using higher resolution equipment such as TEM and SEM which were believed to be the gold standard method of apoptotic analysis, as this instrument can easily differentiate between various cellular deaths pathways (apoptotic or necrotic cells death) through morphological changes. The study demonstrates that apoptosis cells death is the main cause of doxorubicin-loaded calcium carbonate nanocrystal properties; these can be seen by evidence of cells clumping and the formation of apoptotic bodies, membrane blebbing, and the presence of partly degraded apoptotic bodies around the cell cytoplasm as indicated by the SEM analysis. Further morphological analysis was carried out by TEM, which reveals the presence of chromatin condensation, chromosomal DNA fragmentation, cell shrinkage, and nuclear fragmentation. Moreover, TUNEL assay was described in Figure 14; the figure verified that most of the cells undergoes apoptosis by internucleosomal fragmentation of genomic DNA whereas the extent of apoptotic cells was calculated using the apoptotic index (AI) shown in Figure 15; thus, AI is described as the percentage of apoptotic cells and apoptotic bodies within the overall population of total treated cells [27].
